# Repercussions of COVID-19 on the daily lives of women living in a rural settlement

**DOI:** 10.1590/0034-7167-2022-0021

**Published:** 2022-08-08

**Authors:** Jéssica Lima Moura, Harlon França de Menezes, Fernanda Rafaela dos Santos, Rebecca Stefany da Costa Santos, Donatila Cristina Lima Lopes, Janmilli da Costa Dantas, Bárbara Letícia de Queiroz Xavier, Richardson Augusto Rosendo da Silva

**Affiliations:** 1 Universidade Federal do Rio Grande do Norte. Natal, Rio Grande do Norte, Brazil

**Keywords:** Rural Health Services, Women, Rural Health, Pandemics, Coronavirus Infections., Servicios de Salud Rural, Mujeres, Salud Rural, Infecciones por Coronavirus., Serviços de Saúde Rural, Mulher, Saúde da População Rural, Pandemias, Infecções por Coronavírus.

## Abstract

**Objective:**

To understand the repercussions of COVID-19 on women’s daily lives in a rural settlement.

**Methods:**

A qualitative study was conducted in a rural settlement of the Landless Workers’ Movement (MST) in a municipality in Northeastern Brazil between January and March 2021. Forty-eight women participated through semi-structured interviews. The data collected were analyzed by the Collective Subject Discourse method in light of the referential of pandemic processes.

**Results:**

The grouping of the speeches unveiled similar and/or complementary meanings about the coping strategies and the feelings generated due to the pandemic. Four Central Ideas were organized: denial to progressive awareness; Perception of the problem, acceptance, and explanation of reality; Negotiation; and Retrospection/reflection.

**Conclusion:**

The pandemic repercussions are intrinsically related to an inhospitable reality from the perspective of the experience of women daily forgotten, marginalized, and suppressed.

## RESUMO

**Objetivo:**

Compreender as repercussões da COVID-19 no cotidiano de mulheres em um assentamento rural.

**Métodos:**

Estudo de abordagem qualitativa, realizado num assentamento rural do Movimento dos Trabalhadores Rurais Sem Terra, em um município no Nordeste do Brasil, entre janeiro e março de 2021. Participaram 48 mulheres por meio de entrevista semiestruturada. Os dados coletados foram analisados pelo método do Discurso do Sujeito Coletivo, à luz do referencial dos processos pandêmicos.

**Resultados:**

O agrupamento dos discursos desvelou sentidos semelhantes e/ou complementares acerca das estratégias de enfrentamento e dos sentimentos gerados como consequência da pandemia. Foram organizadas quatro Ideias Centrais: Negação ao progressivo reconhecimento; Percepção do problema, aceitação e explicação da realidade; Negociação; e Retrospecção/reflexão.

**Conclusão:**

As repercussões da pandemia estão intrinsicamente relacionadas a uma inóspita realidade da perspectiva da vivência de mulheres que são cotidianamente esquecidas, marginalizadas e silenciadas.

## RESUMEN

**Objetivo:**

Comprender repercusiones del COVID-19 en el cotidiano de mujeres en un asentamiento rural.

**Método:**

Estudio de abordaje cualitativo, realizado en un asentamiento rural del Movimiento de los Trabajadores Rurales Sin Tierra, en un municipio del Nordeste de Brasil, entre enero y marzo de 2021. Participaron 48 mujeres mediante entrevista semiestructurada. Los datos recolectados fueron analizados por el método del Discurso del Sujeto Colectivo, basado en el referencial de los procesos pandémicos.

**Resultados:**

El agrupamiento de los discursos desveló sentidos semejantes y/o complementarios acerca de las estrategias de enfrentamiento y de los sentimientos generados como consecuencia de la pandemia. Fueron organizadas cuatro Ideas Centrales: De la negación al progresivo reconocimiento; Percepción del problema, aceptación y explicación de la realidad; Negociación; y Retrospección/reflexión.

**Conclusión:**

Las repercusiones de la pandemia están intrínsecamente relacionadas a una inhóspita realidad de la perspectiva de la experiencia de mujeres que son cotidianamente olvidadas, marginadas y silenciadas.

## INTRODUCTION

In mid-December 2019, a pneumonia outbreak of unknown cause occurring in Wuhan, China, was reported to the World Health Organization (WHO). Shortly after that, the International Taxonomy Committee identified the etiologic agent as “severe acute respiratory distress syndrome coronavirus secondary to Coronavirus 2” (SARS-CoV-2), a novel coronavirus, and the infectious disease caused by it was named COVID-19^(1)^.

Until March 2022, 449,727,293 cases of infection with the new coronavirus were confirmed, with 6,014,690 deaths caused by the disease worldwide. Brazil ranks third both in the ranking of confirmed cases of deaths, with 29,152,318 accumulated cases and 653,134 deaths up to the same month^(2)^.

These expressive numbers had drastic consequences for all people in the world, especially for individuals who were already in a vulnerable situation before the pandemic. In the case of Brazil, social inequality in all spheres is a reality only intensified by the impacts of the COVID-19 pandemic. In this scenario, this portion of the population that lives in unfavorable social conditions, under the guise of precarious work and low income, suffers from the worsening of inequality potentiated during the pandemic^(3)^.

Among the so-called vulnerable populations, the rural population, especially the settled populations, stood out for its difficulty to access health services, geographical isolation, and structural problems with access to water and basic sanitation. In that regard, this population is significantly affected by infectious and contagious diseases and factors derived from their vulnerability, such as alcoholism, domestic violence, and mental disorders, potentially aggravated due to social isolation^(4)^.

In this perspective, the Brazilian social inequities that overwhelm the rural population make those communities even more vulnerable. While they are socioeconomically disadvantaged and seek informal jobs for subsistence, these individuals cannot achieve social isolation. This situation becomes even more severe for settled women, for they have to work in informal and unhealthy jobs, take care of the family, and take care of themselves and their community.

A pandemic causes several repercussions in people’s daily lives and the socio-historical approach presents itself as capable of explaining the complexity of historically demarcated events, as well as the ideas that circulate among groups and shape their behaviors^(5)^. In this sense, it is necessary to investigate the consequences of the disease in the lives of individuals, in the discussions and decisions of public policies and in the structuring of care, justifying the study.

The relevance of the research is based on the advancement of knowledge about the repercussions of the disease, especially in socioeconomically disadvantaged populations, such as settled women, in order to implement public health actions and policies that are compatible with the demarcations presented socio-historically, ensuring solvability, specificity and uniqueness.

It was assumed that the pandemic causes repercussions that are part of the processes of socio-historical framing of the disease. Thus, this research was guided by the following guiding question: What are the repercussions of COVID-19 on the daily lives of women in a rural settlement?

## OBJECTIVE

Understand the repercussions of COVID-19 on women’s daily lives in a rural settlement.

## METHODS

### Ethical aspects

This study was approved by the Research Ethics Committee of the Federal University of Rio Grande do Norte (UFRN). Its preparation followed the recommendations of the guide to qualitative research Consolidated criteria for reporting qualitative research (COREQ): the 2-item checklist for 3 interviews and focus groups by Red Equator.

### Study design, period and location

That is a descriptive study with a qualitative approach conducted in a rural settlement of the Landless Workers’ Movement (MST), in a municipality of Northeastern Brazil, between January and March 2021. The choice of the research site is justified because it is a rural community with more significant healthcare support in the state. Initially, we requested contact with the leader of the settlement in order to explain the research.

After this request, three visits were made to the settlement, the first with the objective of getting to know the place and its residents, to present the research proposal, and the second and third visits to carry out the interviews. It should be noted that the researchers obtained support from the local Family Health Strategy team, with a community health agent being present on the day of collection.

### Population and eligibility criteria

Inclusion criteria were a woman 18 years of age or older living in the rural settlement. Two researchers recruited the participants personally. Visits were made to the homes of the settled women, inviting them to participate in the research. The person in charge of the settlement provided an open space with three chairs and a table to collect the data.

Data were collected through a semi-structured interview script with open and closed questions based on two theoretical constructs^(5-6)^. This script was divided into four fields: I) Socioeconomic and health data; II) Information on attitudes and strategies to face the COVID-19 Pandemic; III) Daily life of women in a rural settlement during the COVID-19 Pandemic; IV) Repercussions in the face of the COVID-19 Pandemic (psychosocial, socioeconomic, physical-emotional). As a way of improving the conduct of the interviews, before applying the instrument to the women, a pilot test was carried out that did not compose the sample, in order to make the themes and aspects to be discussed during the interview and discussed with the study advisor.

The recruitment of participants was carried out personally by the main researcher and two other authors of the study. An intentional non-probabilistic sampling was performed. The number of participants was delimited, during the fieldwork, by the theoretical saturation of the data^(7)^. Thus, the final sample of this work was, finally, composed of 48 women, who, in addition to providing a satisfactory set of content for interpretation, previously met the pre-established inclusion and exclusion criteria. The interviews were conducted face-to-face, with no refusal or withdrawal at any time.

The interviews were recorded using digital media and fully transcribed by the main researcher and the study advisor, with the interviews lasting, on average, 30 minutes. The information was validated in terms of content by the participants who, at the end of the interview, were asked if they would like to add to the statements.

### Data analysis and processing

The collected data were extracted, coded and grouped into a single corpus of text file, processed by the software Interface de R pour les Analyses Multidimensionnelles de Textes et de Questionnaires (IRaMuTeQ) 0.6 alpha 3, free and open source, through the Similarity Tree and Descending Hierarchical Classification (CHD) methods.

Then, the data were analyzed using the Collective Subject Discourse (CSD) methodology, a method used to create a finite and temporal discourse to extract the common meaning of the interviewees’ speeches^(8)^. Thus, the following steps were: full transcription of the statements; separation of the fragments with meaning; organization of sets of fragments; identification of the Key Expressions (KE) or figurative elements; the combination of the KEs, searching for the reconfiguration of a single discourse composed of subjects’ individual thoughts from the same group. Subsequently, the researchers evaluate and validate the Central Ideas (CI), write and build the discourse synthesis and name them, i.e., give them the CSD title^(9)^.

Thus, the CSD approach assigns meaning as a social actor involving issues of affection, conduct, behavior, cognition and values. Through questioning about what the individual thinks about the problem, what is his opinion about the problem, how he sees it, how he evaluates it and even how this individual feels and positions himself on the problem in question is what we have managed to construct discursive elements treated in research as qualitative data^(10)^. In this sense, as the CSD is built, the system of interpretation of reality by the participants is composed, the relationships established by them in the social context, as well as their behaviors and practices are highlighted.

In addition, the corpus was subjected to interpretation following the logic of Rosenberg’s narrative of pandemic processes. This scholar, from a historiographical and multidimensional perspective, develops an analysis of the perception of social changes generated by diseases, especially epidemics and pandemics, theorizing what he called framing. This process is presented in four characteristic acts, namely: progressive disclosure, management of randomness, negotiation of the public’s response and subsidence and retrospection/reflection^(5)^.

Finally, the use of IRaMuTeQ and the acts of the pandemic processes complemented each other as the integration of statistical methods of the software with the qualitative analysis was allowed, since it was possible to understand the organization of the structure of the reports, the grouping of the set of words, the strength of the connections of this set and the meanings enclosed. The interpretation of what may be behind the lexical similarities and differences is a task that transcends the limits of the software, therefore, the theoretical construct adopted is needed, in this case, the Rosenberg pandemic processes^(11-12)^.

## RESULTS

The participants were between 18 and 60 years old, among them 50% declared themselves brown, 30% black and 20% white, 60% had incomplete elementary school, 40% were married, 50% had more than 2 children, 20% lived with elderly relatives, 60% had houses made of wood and 70% lived with an income below the minimum wage.

Women reported concerns generated in the face of the pandemic such as: lack of work; difficulty supporting the family due to lack of resources; uncertainty about the future; risk of contamination; fear of death; cancellation of children’s classes; as well as referring to the actions forged to face the pandemic, among which the adherence to protection and prevention measures, the solidarity actions between neighbors of the community and the exercise of faith.

Opportunities in experiencing the pandemic were also revealed, such as the chance to learn new things and reinvent themselves despite everything, which provided the renewal of hope for a better future.

The grouping of the settled women’s speeches reveals similar and/or complementary meanings about the coping strategies and feelings resulting from this action. The emergence of those strategies and emotions was organized through four Central Ideas of analysis.

### Central Idea A: Act 1 - Denial to progressive awareness

This first CSD characterizes the disbelief in the potential destruction of the pandemic event. In the women’s speech, it was evident the negationism about the reality of the pandemic and the prevention actions.

*At first, we did not believe in the seriousness of this disease, in fact, no one believed that this pandemic would reach the settlement, but it must be recognized that it arrived and killed many people, because the government was not prepared to deal with it. Everyone here denied this disease, but now it is necessary to recognize that it is present and it is necessary to recognize that anyone can die from it. Despite denying and not believing in this disease, I even thought it was a little flu, but it arrived here in the settlement and today we recognize that it made many people sick and poorer, needier. When this pandemic emerged, no one really believed that so many people would die, so no one cared about the prevention that was recommended on television. I think even the government denied it for not being prepared to face this pandemic. Nobody thought it was that serious, let alone that it would reach the settlement. Before, I only heard about it on the radio and the television, and now, I see it in real life; it’s in front of me, it’s my neighbor, people in my family are dying, and then I realize that the situation is too serious, and the problem is very much real.* (CSD of settled women in Northeast Brazil during the COVID-19 pandemic)

### Central Idea B: Act 2 - Problem’s perception, acceptance, and explanation of reality

The settled women’s speech goes through a transformation due to the increase in confirmed cases and deaths from COVID-19. Negationism gives way to fear of death and uncertainty about the future. There is an attempt to explain the phenomenon, and the concern with the future repercussions is evident.

*After seeing so many people dying and no ICU vacancy, I have to recognize that this disease is very serious. I didn’t really appreciate what was happening, I thought it was some flu. Only now do I realize that it is a very serious disease. Before, we only watched it on television, but today we realize that COVID-19 has already arrived here in the rural settlement and is already affecting our entire community. We also realize the government’s inability to tackle this disease. It seems that we are living a nightmare that never ends. Living a moment like this, of a pandemic that is killing a lot of people, every day, is desperate and scary. Everyone needs to accept that it is a disease that kills. I have a lot of fear and anxiety. I am afraid of dying and leaving my children abandoned. I live insecure because the authorities, politicians and health personnel cannot stop this pandemic. I hear that there are many people dying all over the world and here I saw people who went to the hospital and did not come back because the government is not managing to face this disease, so it is only up to us to do our part with prevention measures. When this pandemic started, everything that was of service stopped. There was no work and I had no way to support the family. That’s when I realized that this disease was really very serious. With this disease I became unemployed, I lost the cleaning I used to do. I was fired because they wouldn’t let me take my son with me to work, and they’re out of class because of the closing of schools and day care centers. Then I realized that it was very serious. We can see that it is a very serious disease because Brazil is not managing to face this pandemic, let alone the politicians here. Here in the settlement we have many religious values. We are all asking God for the cure of this disease. I hope I can witness a cure for this disease, my values are based on my faith in God, and I believe we will overcome COVID. When we have our values in God, we believe in God, with our faith and hope, we accept His will, and it is easier to overcome all of this.* (CSD of settled women in Northeast Brazil during the COVID-19 pandemic)

### Central Idea C: Act 3 - Negotiation

Motivated by the fear of contamination, the settled women expressed their actions of confrontation. Textual fragments demonstrate the emergence of a collective action culminating in making crucial decisions for confrontation and the feelings generated by adherence to sanitary measures.

*Despite so much pain and difficulty, you have to keep facing this pandemic. Each of us needs to work hard and do our part, wearing the masks, lots of alcohol to clean everything and always wash our hands and also not forget that you can’t cluster. We all need to make an effort, each doing their part. As the government is not being able to face this disease, we have to follow the prevention measures to survive. Either follow or die. And every day I hear different opinions. One doctor orders chloroquine, another orders ivermectin. Each one says something different, but the health team came here, consulted with us and explained everything. The important thing is to follow the prevention measures that the health team recommended to face this disease. Anyone can contract this disease, whether black, bank, rich, poor, young or elderly, so the real way is to recognize the danger of this disease and carry out preventive measures. When you have this disease and need to be isolated, anyone becomes restless, afraid, insecure, and also ends up feeling a lot of anxiety, you can’t help but be nervous and apprehensive, seeing people dying here in the settlement every day. Even so, if someone has the disease, they need to follow the guidelines that scientists are always talking about on television shows. I’m insecure about the future. It’s all very unstable and I haven’t slept well since all this started. I live anxious, insecure and stressed, wishing this pandemic would end soon. I don’t see any improvement in the future. It’s all uncertain. In addition, there is uncertainty in the cure and the risk of any of us contaminating ourselves and dying is great. We have tried to follow the guidelines of the Nurse who makes the home visit here in the settlement. It is necessary to prevent. Nobody can be careless. We have also tried to pray a lot to reduce anxiety. The obligation of having to stay at home isolated brought a lot of anxiety, stress and crying among women here in the settlement, but it is necessary to follow this recommendation from health professionals. I feel very lonely, as I have lost contact with my mother and sisters, who are already elderly. It is very difficult to be in social isolation all the time and not be able to leave the house, mostly because I helped a lot in the church and my community, and I miss that a lot.* (CSD of settled women in Northeast Brazil during the COVID-19 pandemic)

### Central Idea D: Act 4 - Retrospection/reflection

Since we conducted the study during the COVID-19 pandemic, the framework of this phase of the epidemic event was not totally consolidated. However, it is possible to observe in the discourse textual fragments capable of elucidating the meaning extracted from the experience of the settled women regarding the pandemic; and the “lessons” learned from living in this context can also be noted.

*I think that living in this pandemic and facing death has transformed my life, my ideas, all of this has brought me a lot of learning, made us understand that we are very fragile, capable of overcoming everything and gave me more encouragement to continue wanting to live, because the pandemic increased my faith in God. The great lesson that remains for everyone is that human beings can overcome everything, doing good and helping others. It is at this moment that everyone needs their neighbor, their family, their community. This disease came to make us more human and help others. Everyone also came to realize that the government can’t face this disease. I think it made everyone reflect on how the government and health professionals were unprepared to deal with this disease. This year was lost and this brings me a lot of sadness and discouragement, I think that when this pandemic is over, we will be stronger women, more warriors, because the only positive point I see with this pandemic is the opportunity to rethink life. It is rethinking that life is more valuable than money and that it is possible, even with this tragedy, to do new things and always learn new things. I learned to take advantage of this difficult time and, despite everything, I am very happy and grateful for the help I have received from friends, neighbors and the church. I think living all of this has renewed my faith, hope for the future and trust in God. I started to believe a lot in God and hope for a better future; I became more human, willing to help my community more, and then the sadness that was taking over me passed. After all, life is now, and the future is now.* (CSD of settled women in Northeast Brazil during the COVID-19 pandemic)

**Figure 1:**
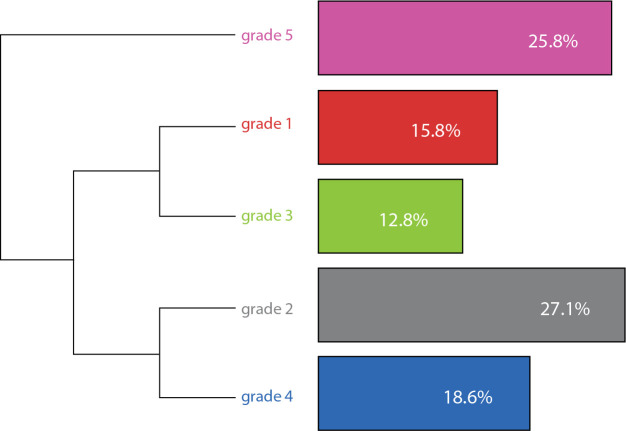
Descending Hierarchical Classification Dendrogram, organized based on the IRAMUTEQ software, Natal, Rio Grande do Norte, Brazil, 2021

When reading the textual corpus of speeches obtained through the software, it was possible to extract five interrelated and divergent semantic classes by Descending Hierarchical Classification through the Reinert Method (Figure 1).

In class 1 it is possible to observe the prevalence of terms related to the phenomenon of reflection. In class 2 it is possible to observe the relationship of the terms with the phenomenon of negation, predominant in the speeches. In class 3, there is a lack of classification, generally correlated to terms that do not fit into the other categories. In class 4, reflection is assimilated as a phenomenon correlated to the words evoked. Finally, class 5 is tangent to the phenomenon of acceptance.

**Figure 2:**
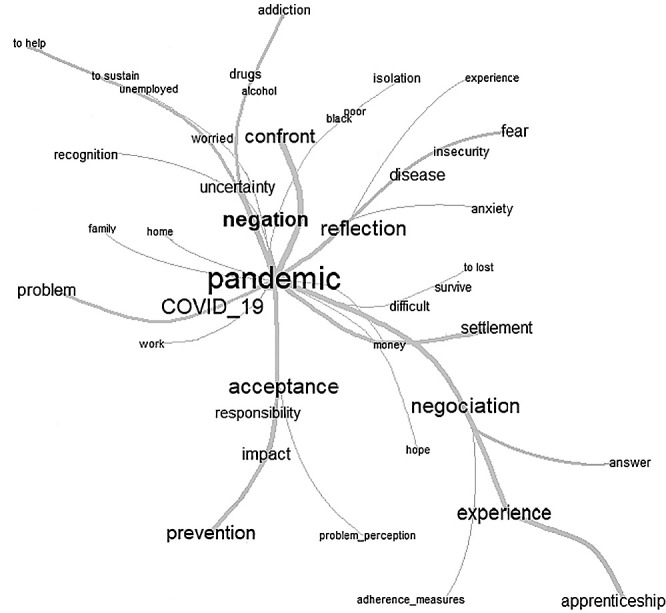
Tree of Similarity about the repercussions of COVID-19 on the daily lives of women in a rural settlement extracted from the IRAMUTEQ software, Natal, Rio Grande do Norte, Brazil, 2021

For the establishment of the analysis of similarity and given the extensive quantity of words extracted from the interviews, as well as the need to answer the research question with terms of greater prevalence, repetitions equal to or greater than ten were considered, making it possible to observe the similarities between the words, both the degree of correlation and the comparison of frequency through lines and sizes graphically represented (Figure 2).

In the center, it is possible to observe the word “pandemic” and “covid_19”, which infers that they were present at all times and with great intensity. Five branches of greater thickness depart from the center, which contain “negation” and “reflection” in the upper portion and “negotiation” and “acceptance” in the lower margin.

The above graphic provision infers that Covid-19 in the speeches present in the first act (denial) was the predominant feeling of “uncertainty”, given the presence of the segments “concern”, “unemployment” and “sustenance” and their higher ramifications with the terms “addiction”, “alcohol” and “drugs”. This provision reflects, in addition to the common aspects of the chaos caused by the Covid-19 pandemic, plus the circumstances of extreme vulnerability experienced by the homeless.

The lower left branch that is marked by the presence of the word “acceptance” and its derivations “perception_problemas”, “responsibility”, “impact”, “adherence”, “measures_prevention”. Which infers to say that after the first act, and contrary to it, the acceptance phase allows the redirection of thoughts to a condition of alert/caution about the risks that involve a certain circumstance, confirming the presence of the second act in the analysis of similitude of that study.

The third act is evidenced in the upper left branch from the term “reflection” in which the predominance of the feeling of “fear”, “anxiety”, “insecurity”, “experience” and “illness” is marked. By analyzing these words together, it is possible to infer that, at this stage, feelings of concern regarding the present scenario of Covid-19 predominate, essentially because of the bad experiences of the interviewees so far.

Finally, the fourth and last act, the “negotiation” followed by the ramifications “adherence to measures”, “learning” and “response” evidence the phenomenon of confrontation naturally promoted in this phase, which for the most part, tend to have positive results in the individual actions and adherence to preventive practices.

## DISCUSSION

Coping with a pandemic encompasses individual and collective spheres of action that permeate the actions of the State and citizens, joining forces and knowledge in order to minimize the damage resulting from the disease. The CSD very clearly demonstrates the aspects relevant to coping with the COVID-19 pandemic from the perspective of the settled woman, from those applicable to the State, such as the action of health teams, to individual actions for the exercise of solidarity and faith.

In the past, rural communities worldwide have been heavily affected by other pandemics. Today, in the context of COVID-19, it is clear that there are persistent infrastructure issues in those areas that have not been addressed, much less resolved. Furthermore, there is a disparity in the demographics of the rural population, which is older, and that, associated with a lower quality of life, reflects directly in worse overall health indicators. With rural populations having less access to those resources, the death rate from COVID-19 may increase. These observations suggest a lack of preparedness by states for pandemics in the rural context; therefore, the global spread of COVID-19 may significantly impact rural communities to a greater extent^(13)^.

Thus, it is evident that the pandemic generated in our environment an economic decline in individuals and families since among the most effective measures to control the disease are social distancing and quarantine. The impact of the strategies on low-income populations is noticeable throughout the CSD because those populations have to choose between obeying the guidelines for prevention purposes - and staying at home/isolated from social life - or going out to work to provide for themselves and their families. This pre-pandemic reality will undoubtedly continue when the pandemic is over^(14)^.

Thus, when we apply a “zoom” from the social point of view between the so-called vulnerable populations, we identify an even more socially vulnerable portion, that is, women, heads of families, poor, black, marginalized and settled, as is the focus of this study. Allocated in underemployment, many of them lost their jobs as a result of the pandemic control strategies, such as quarantine and lockdown, and that is why these women adhered to informal or autonomous work.

However, besides increasing the risk of contamination, this work could not guarantee these women sufficient financial resources to meet their basic living needs and those of their families. The reality described in the CSD corroborates a study demonstrating that women are more exposed to the risk of infection by the new coronavirus given the pre-existing social inequality to which they are subjected historically^(15)^.

On the other hand, for the chiefs of families, female providers of their homes and children, there was no other way out but to search for work, waging an actual battle for survival. That converges with the history of women’s struggle, constantly transgressing standards to pursue the guarantee to have the right to their own body, their health, vote, and, especially, their freedom^(16)^. Thus, considering the dimension of the informal job, one realizes that not obeying the maxim “stay at home” is also a form of struggle for survival and coping^(17)^.

From this perspective, the feelings triggered as a consequence of the negative repercussions of the pandemic were exposed throughout the CSD, especially in the second act, among which anxiety, fear, anger, sadness and feelings of powerlessness stand out. This occurs as a consequence of the acceptance of reality and perception of the negative repercussions that the pandemic has brought to the settlement.

Fear is a potentiating feeling of anxiety in the most vulnerable population, especially in the rural population, due to geographic isolation, the difficulty in accessing education and professionalization and the scarcity of economic opportunities and, therefore, it is estimated that the number of people with affected mental health tends to be greater than the number of people infected with the disease^(18)^. However, what aggravates this scenario of fear provided by COVID-19 is the manifestation of uncertainty about the end of the pandemic and, mainly, about the future.

In this sense, social isolation can be pointed out as one of the main factors responsible for the emotional vulnerability of individuals and it is evident that the feeling of fear is a natural and even physiological reaction to a context of serious threat to life^(19)^. There is evidence that the exacerbation of these feelings compromises the mental health of the individual, especially when they are touched by a close connection to social determinants such as poverty, employment, education, housing, urbanization and sexual discrimination, which corroborates a study carried out that identified greater levels of stress, anxiety and depression in women, demonstrating that the psychological impact of the pandemic may be greater in this group^(20-21)^.

Considering this context, it is known that religion and spirituality - which have been part of human experience since the oldest civilizations - are also constituted as a dimension of care. Religion and spirituality promote the exercise of faith, which, in turn, is an important sociocultural element capable of directly intervening in all aspects of human life, bringing to light the personal dimension of the meaning of life^(22)^.

In this sense, in order to minimize the extent of these feelings inherent in the pandemic context, actions of solidarity and the exercise of faith are fundamental. In the analysis of the results, it is possible to identify the relationship between spirituality, solidarity and faith as coping strategies for the settled woman in the face of the pandemic, as well as a way to generate resignation to explain and understand the pandemic, which is evident in the second act of the CSD. The direct action of the authorities and religious institutions on the daily lives of these women generated a feeling of hope in better days, strengthening the face of adversity. The findings corroborate a study carried out with another vulnerable population, the elderly, where spirituality was able to promote emotional support, support and protection^(23)^.

The first CSD deals with the denial of the severity of the pandemic, as well as its potential for lethality and destruction. It is noticeable, throughout the speech, that this denial did not consist only of a mere “denial of the problem”, but also and, consequently, a refusal to adhere to prevention measures. This fact can be attributed to the total disarticulation on the part of the Federal Executive Government to health authorities on a global scale, and to state and municipal governments, which ended up delaying the country’s ability to respond to the health crisis created by COVID-19^(24)^.

Regarding adherence to prevention measures, the third CSD expresses very emphatically how these were highlighted by the settled women. The use of a mask, frequent hand hygiene, the use of gel alcohol and respect for social distance, avoiding agglomerations, appeared throughout the CSD, more precisely in the third act, as coping strategies with the aim of mitigating the advance of the disease to which the settled women adhered.

However, even aware of the severity of the disease and how they could protect themselves, it is clear that the limitations imposed by poverty, such as the difficulty of full access to health services, basic sanitation, and low purchasing power to obtain items of hygiene and mask, made it difficult to adhere to these measures. It is known that socioeconomic and cultural aspects have a direct influence on adherence to prevention measures, and it is up to governments to institute measures to facilitate this process^(25)^.

Even so, the fundamental role of Primary Health Care (PHC) professionals is evident, especially community health agents (CHA), in the dissemination of protection measures and in encouraging adherence among settled women. The role of CHAs in fighting epidemics has already been addressed in the literature, and these professionals are the main responsible for the permeability of the actions recommended by the health authorities, supporting educational activities in order to promote the awareness of the population^(26)^.

From this perspective, even though there is sufficient evidence regarding the effectiveness of prevention and protection measures in reducing the spread of the new coronavirus, the sustainability of these measures depends mainly on the implementation of public social protection policies that guarantee vulnerable populations the chance to adhere to them. Although this population is aware and wants to incorporate such protective habits against COVID-19, the precarious situation resulting from the social inequities in which they live makes this adherence impossible.

In this sense, the solidarity and fraternal relationship of the participants of this study with the rural settlement community was highlighted in the second act of the CSD, being placed as essential elements for the maintenance of the subsistence of these women and their families. However, it is worth noting that the principle of solidarity should not only be incorporated into actions of an individual nature, but also and mainly in actions of a collective scope, with the incisive participation of health authorities in the elaboration of public policies in order to promote the well-being of people, cooperating for the realization of the right of all people to their dignity, including exclusivity in decision-making^(27-28)^.

The multiprofessional work with an interdisciplinary focus, where Nursing can be highlighted, allows the health needs of this population to be fully met and evaluated. Multiprofessional actions reinforce common goals and promote an impact on the experiences of people who need more health care.

### Study limitations

The limitations of this study are evident in the time frame, given that, at the time of carrying out this investigation, the pandemic is still not under control and the negative repercussions, as well as the number of cases and deaths, continue to increase. Furthermore, in view of the immense regional differences that characterize Brazil, it is necessary for studies like this to be reproduced among women from rural settlements in other regions of the country, to investigate the behavior of the pandemic, as well as its repercussions in similar contexts and at the same time so different.

### Contributions to the Area of Nursing and Public Health

The contribution of this study is to recognize the consequences that the pandemic has caused in the daily lives of women in the rural context. The social movement in question reinforces political issues and fragile health needs, where the implementation of assistance measures for these women becomes preponderant.

## CONCLUSION

The study allowed us to understand that the repercussions of COVID-19 in women’s daily lives in a rural settlement went through the following acts: denial to progressive awareness; problem’s perception, acceptance, and explanation of the reality; negotiation; and retrospection/reflection. The women revealed denialism about the reality of the pandemic, as well as about prevention actions. However, negationism gives way to fear of death and uncertainty about the future. Finally, collective action culminates in making crucial coping decisions and feelings and “lessons” generated by adherence to sanitary measures.

In this sense, the reflections and meanings extracted from this study bring to light an inhospitable reality through the perspective of the experience, experience and voice of women who are daily forgotten, marginalized and silenced.

Therefore, the present research highlights the importance of more studies of this nature being carried out so that the course of the disease is studied in different contexts and feasible and adequate measures are finally taken. It is hoped that the speeches coming from the protagonists are capable of promoting a change of thought that motivates the struggle for a more just, equitable and equal society for all.
